# Active implementation of low disease activity state as a treatment endpoint in childhood-onset systemic lupus erythematosus in routine practice is both feasible and associated with better outcomes

**DOI:** 10.1007/s10067-024-07101-4

**Published:** 2024-08-30

**Authors:** Ruby Gotch, Yumna Ahmed, Robert Wilson, Ellie Hawkins, Coziana Ciurtin

**Affiliations:** 1 Department of Adolescent and Young Adult Rheumatology, University College London NHS Foundation Trust, 250 Euston Road, London, NW1 2PG UK; 2 Centre for Adolescent Rheumatology Versus Arthritis, Division of Medicine, University College London, Rayne Building, London, W1CE 6JF UK

**Keywords:** Childhood-lupus low disease activity — cLLDAS, Childhood-onset systemic lupus erythematosus — cSLE, Feasibility study, Treat to target — T2T

## Abstract

**Introduction:**

Treat-to-target (T2T) strategies aim to facilitate tight disease control to improve outcomes. No previous studies evaluated prospectively the feasibility and impact of the T2T strategy in routine practice in childhood-onset SLE (cSLE).

**Methods:**

Adolescents and young adults (AYA) with cSLE were recruited for T2T implementation from a large tertiary centre over a period of 6 months and followed up at least twice over a prospective period of 12 months.

**Results:**

During Oct 2022–April 2023, 135/162 (83.3%) AYA with cSLE had disease scores evaluated at their routine appointment to enable inclusion in the study, and 122/135 (91.2%) had their disease assessed, and a suitable treatment target agreed and documented at each routine clinical appointment over the 12 months prospective follow-up. T2T strategy led to improved disease control at 12 months: more AYA with cSLE achieved clinical remission off steroids (4.1% vs. 10.7%, *P* = 0.048), or minimum childhood-lupus low disease activity (cLLDAS) (81.9% vs. 91.8%, *P* = 0.022). Achieving minimum cLLDAS for longer than 3 months was associated with reduced damage accrual (HR = 1.7; 95%CI = 1.1–2.5; *P* < 0.0001) at 12 months.

**Conclusion:**

T2T strategy implementation was achievable and associated with improved cSLE control. Spending at least 3/12 months in cLLDAS led to less damage accumulation.

**Table:** 

**Key Points**
• *This is the first large prospective study in AYA with cSLE to evaluate the impact of active T2T implementation in routine practice.* • *T2T strategies were feasible to implement in 122/135 (91.2%) AYA with cSLE in routine practice.* • *The T2T approach was associated with improved disease control and decreased damage accrual at 12 months.*

## Introduction

Systemic lupus erythematosus (SLE) is an autoimmune rheumatic disease associated with complex immune system dysregulation perpetuating chronic multi-system inflammation, with an unpredictable clinical course, potentially leading to irreversible damage and significant co-morbidity burden [1]. Children and young people who develop clinical symptoms before age 18 are classified as having childhood-onset SLE (cSLE), which accounts for approximately 10–20% of all SLE patients. The disease is frequently diagnosed at the time of puberty (with the exception of monogenic SLE, usually diagnosed before age 5). In the UK, the age at cSLE onset is 12.4 years, which coincides with the age at menarche [2]. The disease is very heterogeneous in terms of clinical presentation and severity and has an overall female predominance (girls to boys = 4.5:1) [3] and a global incidence of 0.5–6 per 100,000 [4].

Despite being a rare condition, cSLE is more severe than adult-onset SLE and requires more intense immunosuppressive treatment [3, 5]. cSLE is also associated with a high co-morbidity risk from early life [6], with 20.4% of cases already having irreversible organ damage at 1-year follow-up [7]. Despite this, the evaluation of long-term outcomes and comorbidity risk later in life are hampered by a lack of long-term follow-up studies from childhood into adulthood or the availability of age-appropriate validated outcome measures for assessment of comorbidity risk [8], which all pose challenges in ensuring optimal life-long management of cSLE [9].

Treat-to-target (T2T) strategies are focused on proactive and tailored management of disease activity to minimise damage risk and improve long-term outcomes in SLE across ages. Several cohort studies validated T2T endpoints in adult-onset SLE in relation to improved outcomes [10, 11]. Recent efforts have been made to develop similar T2T strategies in cSLE using T2T outcome definitions adapted from those validated in adult SLE. They advocate for remission, and if not achievable, for low disease activity (LDA) as treatment targets in both cSLE [12, 13] and SLE [14].

T2T outcome definitions in cSLE include childhood lupus low disease activity state (cLLDAS), cSLE clinical remission on-corticosteroids (cCR), and cSLE clinical remission off-corticosteroids (cCR-0) [13, 15]. Retrospective evaluation of treatment target attainment in cSLE in the large UK JSLE cohort found that 67% of individuals achieved LLDAS [16] after a median duration of 18 months [7], although this was estimated retrospectively in a selected cSLE cohort recruited for research purposes and followed up for a median duration of 2 years (0.4–4 years).

The suitability and impact of active prospective implementation of T2T strategies in routine clinical practice in a large cSLE cohort with longer duration follow-up have not been evaluated before. Despite increasing interest in the T2T approach in cSLE, the studies published recently relied exclusively on retrospective data analyses or analysed data from prospective cSLE cohort studies which were not specifically designed to evaluate T2T strategies.

Our hypothesis was that T2T strategies can be implemented in routine clinical practice in a large cohort of adolescents and young adults (AYA) with cSLE (actively involved in discussing and agreeing a treatment target with their clinicians) and that T2T strategies can lead to improved disease control over 12-month routine follow-up.

This study aimed to assess the feasibility of agreeing on and documenting a treatment target in a large cohort of AYA with cSLE and explore the impact of setting cLLDAS as a therapeutic target on disease states over a 12-month routine follow-up period.

## Methods

We used a prospective real-life cSLE quality improvement evaluation cohort study design to address the aims stated above. The study included two phases: a recruitment and an evaluation phase (not phases), as required by a quality improvement study (QIP) design.

**Fig. 1:**
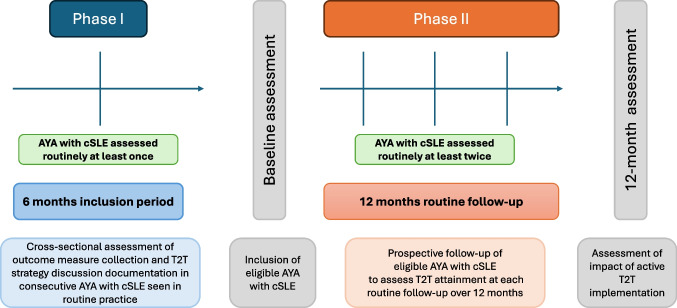
Study design. Legend: AYA, adolescents and young adults; cSLE, childhood-onset systemic lupus erythematosus; T2T, treat to target

The project phases are detailed in Fig. 1, which includes the study design and timepoints at which AYA with cSLE were routinely assessed for inclusion, and throughout the follow-up period.

The first phase of the study was a 6-month cross-sectional evaluation of the feasibility to implement and systematically document routine outcome measures in AYA with cSLE. Each potential participant was assessed for eligibility against the inclusion criteria (see below). At the end of this phase, only AYA with cSLE and complete data collection, including disease activity evaluation as well as treatment target assessment and documentation, were included. Data collected at inclusion was designated as participants’ baseline assessment (see Fig. 1).

The second phase of the study was a 12-month prospective evaluation of disease control against the agreed cSLE target between the baseline assessment and the final timepoint (last routine clinic appointment during the 12-month study follow-up period). Only AYA with cSLE and complete disease activity, treatment target assessments and documentation at least at two time points over a prospective period of 12 months of routine follow-up were included.

### Inclusion criteria

AYA with cSLE were classified based on the Systemic Lupus International Collaborating Clinics (SLICC) 2012 criteria [17] and/or the European Alliance of Associations of Rheumatology/American College of Rheumatology (ACR) 2019 criteria [18]. Data about the cumulative organ involvement, serological markers, cumulative treatment, including steroid dose as well as the paediatric version of the British Isles Lupus Assessment Group (pBILAG) score [19], Systemic Lupus Erythematosus Disease Activity Index (SLEDAI)-2 K score [20], and the paediatric version of the SLICC/ACR Damage Index score (pedSDI) [21], as well as physician global assessment on a 0–3 visual analogue scale (PGA), were collected longitudinally at each routine appointment.

There were no exclusion criteria as all AYA with cSLE reviewed consecutively in routine clinical practice were assessed for potential inclusion in this project. This strategy mitigated against the risk of additional selection bias, although we appreciate that AYA with cSLE who failed to attend regular routine clinical appointments have not been captured within this analysis.

AYA with *disease control in target* were sub-classified as in either LLDAS, cCR, and cCR-0, as per proposed definitions [15], as well as in *complete remission*, defined as SLEDAI-2 K = 0 on a maximum of 5 mg or 0.1 mg/kg prednisone daily or equivalent) and *complete remission off steroids*, defined as SLEDAI-2 K = 0 on no steroids. AYA with *disease control not in target* were classified as having mild (SLEDAI-2 K > 4 and < 6), moderate (SLEDAI-2 K ≥ 6 and < 10), and high disease activity (SLEDAI-2 K ≥ 10).

The two clinicians routinely assessing AYA at routine clinical appointments have received training regarding validated outcome measures and T2T strategy implementation which was delivered as part of the QIP project. 

### Statistical analyses

Data were analysed using R software version 4.2.2 (RRID:SCR_001905). Distributions were visualised using density plots. Formal Shapiro–Wilk normality testing was also performed to assess normality. Paired two-sided Mann–Whitney *U* tests or Student’s *t*-tests were applied to test differences between two groups as appropriate, depending on the data distribution. Univariable and multivariable interval-censored survival regression models were used to compare LLDAS (including those on remission) versus those not on LLDAS for at least 3/12 months.

This study was a prospective cohort study rather than a research project aiming to identify the impact of T2T strategies on the proportion of individuals with cSLE achieving minimum LLDAS at 12 months compared to baseline. However, in support of our project design robustness, a preliminary sample size calculation was undertaken, which showed that we needed to include at least 115 individuals to be able to detect with 90% confidence and 80% power, a 10% improvement in the proportion of AYA with cSLE achieving LLDAS after 12 months of active T2T strategy implementation.

## Results

During 1st Oct 2022–1st April 2024, 135/162 AYA with cSLE routinely evaluated in the clinic have been eligible for inclusion. They had the following characteristics: mean age of 26.5 ± 5.1 years; mean disease duration of 13.5 ± 4.8 years; 85.1% females, with almost an equal split between White (40, 29.6%), Black African/Afro-Caribbean (38, 28.1%), and Asian ethnic background (40, 29.6%), and 12.5% of other ethnicity. Details about the cumulative clinical and serological features, classification criteria fulfilled, treatments and disease activity scores, and damage are provided in Table 1. The majority of AYA with cSLE had a SLEDAI ≤ 4 (126, 93.3%), while 7/135 (5.1%) were experiencing moderate or severe flares at inclusion. More than one in three AYA with cSLE already had damage (50, 37%) (Table 1).

**Table 1:** Demographic and disease characteristics of the initial cSLE cohort assessed during the first phase of the prospective study

	JSLE cohort
**Total number**	135
Female to male	115:20
Median age (years)	26.5 ± 5.1 years
Mean disease duration ± SD (years)	13.5 ± 4.8 years
Median age at onset ± SD (years)	12.3
Ethnicity (%)	
White	40 (29.6%)
Black	38 (28.1%)
Asian	40 (29.6%)
Other	17 (12.5%)
Cumulative clinical features	Number (%)
Renal involvement	60 (44%)
Constitutional involvement	96 (71.1%)
Neuropsychiatric involvement	24 (17.8%)
Mucocutaneous involvement	116 (86%)
Musculoskeletal involvement	89 (66%)
Haematological involvement	101 (75%)
Cardiorespiratory involvement	21 (15.5%)
Gastrointestinal involvement	5 (3.7%)
Ophthalmic involvement	0 (0%)
Cumulative serological features	Number (%)
ANA positivity ever	135 (100%)
Current ANA positive	113 (83.7%)
Anti-dsDNA positivity ever	72 (53.3%)
Current anti-dsDNA positivity	54 (40%)
APS screening positive twice (ever)	11 (8.1%)
Cumulative classification criteria fulfilled	Number (%)
2012 SLICC classification criteria	135 (100%)
2019 ACR/EULAR classification criteria	132 (97.7%)
Current treatment (unless specified otherwise)	Number (%)
None	10 (7.4%)
Current B-cell targeted therapy	15 (11.1%)
B-cell targeted therapy ever	45 (33.3%)
Hydroxychloroquine	115 (85.2%)
Methotrexate	14 (10.3%)
Azathioprine	27 (20%)
Mycophenolate mofetil	74 (54.8%)
Cyclophosphamide in the past year	5 (3.7%)
Cyclophosphamide ever	26 (19.2%)
Current prednisolone dose ≤ 5 mg daily	73 (54%)
Current prednisolone dose > 5 mg but ≤ 7.5 mg/day	6 (14%)
Current prednisolone dose > 8 mg daily	36 (26.6%)
Not on prednisolone	20 (14.8%)
Disease activity/damage scores	Number (%)
Average SLEDAI, *n* = 135	1.6 (0–18)
SLEDAI = 0	60 (44.4%)
SLEDAI ≤ 4	66 (48.8%)
SLEDAI = 5–9	6 (4.4%)
SLEDAI ≥ 10	3 (2.2%)
PedSDI ≥ 1	50 (37%)
PGA VAS = 0	90 (66.6%)
PGA VAS ≤ 1/3	36 (26.6%)
PGA VAS > 1	9 (6.65)

*ANA*, antinuclear antibodies; *dsDNA*, double-stranded DNA; *SLEDAI*, SLE Disease Activity Index; *PedSDI*, Paediatric SLE Damage Index; *PGA*, Physician Global Assessment; *SLE*, systemic lupus erythematosus; *VAS*, visual analogue scale

### Implementing routine outcome measure collection in clinical practice is feasible

Only 13/135 (9.8%) AYA with cSLE had incomplete assessments or no therapeutic target discussed/recorded when assessed during the 6-month inclusion period. The SLEDAI-2 K, (pedSDI), and PGA were recorded in 122/135 (91.2%) AYA with cSLE, and the (pBILAG) score was recorded at every assessment only in 92/135 (68.1%) of clinical letters (*P* < 0.00001). At the inclusion in the study, the disease activity scores had a skewed distribution, as expected, with the large majority of AYA having good disease control: median SLEDAI-2 K = 0 (IQR = 2), mean SLEDAI-2 K = 2 ± 2.79, median global pBILAG score = 0 (IQR = 1), and mean global pBILAG score = 0.96 ± 2.97. The median PedSDI score was 0 (IQR = 1), with 47 (38.5%) overall having already acquired damage: mild damage (PedSDI = 1 or 2) in 37/47 and severe damage (PedSDI ≥ 3) in 10/47 AYA with cSLE.

### Agreeing with AYA with cSLE on a treatment target is achievable

In total, 122/135 (90.4%) had a therapeutic target initially agreed and assessed against at least at two, and 82/122 (67.2%) at least at three different time points over 12-month routine follow-up (338 routine clinical assessments for the whole cohort). The reasons for not agreeing on a target in 13/135 cases were the following: 5/13 (38.5%) AYA were experiencing cSLE flares at baseline and setting a target was deemed not feasible, while in 8/13 (62.5%) cases, the assessment against a feasible treatment target was not consistently documented, potentially because of time constraints. The definitions and proportions of various disease activity states at the clinical appointment at which the target was agreed (baseline) compared to the last follow-up (at 12 months) are detailed in Table 2.

**Table 2:**
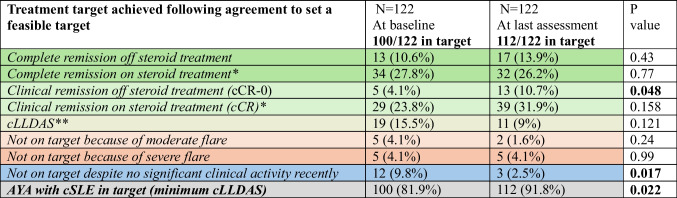
Assessment of disease states at the time point of agreeing a treatment target (baseline) and at 12-month routine follow-up (*P* < 0.05 was considered statistically significant)

*AYA*, adolescents and young adults; *cLLDAS*, childhood lupus low disease activity state

^*^Equivalent of prednisolone dose ≤ 0.1 mg/kg daily, maximum of 5 mg daily

^**^Equivalent of prednisolone dose ≤ 0.15 mg/kg daily, maximum of 7.5 mg daily

Complete remission was defined as SLEDAI-2 K score = 0

Clinical remission was defined as clinical SLEDAI-2 K score = 0

For remission states, stable treatment with antimalarials and stable conventional or biologic immunosuppressive treatment were permitted as per published definitions

cLLDAS was defined as SLEDAI-2 K ≤ 4, no major organ involvement, no new cSLE features; physician global assessment ≤ 1 (0–3 scale); equivalent of prednisolone ≤ 0.15 mg/kg daily, maximum of 7.5 mg daily, no intravenous methylprednisolone; standard maintenance immunosuppressive drugs/biological agents

Moderate flare was defined as SLEDAI-2 K ≥ 6 but < 10

Severe flare was defined as SLEDAI-2 K ≥ 10

### Setting cLLDAS as minimum therapeutic target in cSLE was associated with improved disease control after 12-month follow-up

Out of 338 independent assessments across the whole cohort, 295 were spent on target (86.9% of the time); 45/122 AYA with cSLE improved their disease control (including 39/122 (31.9%) who achieved even a better target and 14/122 (11.5%), previously active or on a higher dose of steroids than clinically indicated, who achieved the agreed target). After 12 months, a significant improvement in the proportion of AYA achieving cCR-0 (4.1% vs. 10.7, *P* = 0.048) and a decrease in the number of AYA not on target (9.8% vs. 2.5%, *P* = 0.017) have been achieved (Table 2). Seven AYA with cSLE (5.7%) experienced moderate/severe flares**,** and another three (2.5%) did not achieve their set target as they have not been able to decrease their steroid dose or had ongoing disease activity. There were no statistically significant predictors for achieving cLLDAS as the minimum target versus flaring over the 12-month period investigated due to the low number of AYA who flared.

We explored various correlation analyses between damage scores and clinical predictors and found only a positive correlation between the pedSDI score and cumulative steroid dose (median dose = 615 mg) over the 12-month period of the study (ρ = 0.37, *p* = 0.04).

Achieving minimum cLLDAS for longer than 3 months was associated with reduced damage accrual (HR = 1.7; 95%CI = 1.1–2.5; *P* < 0.0001) and flare risk (HR = 1.6, 95% CI = 0.98–1.4; *P* = 0.06) over the 12-month study follow-up duration, but the last was not statistically significant.

Actively implementing a T2T strategy led to an increase in the proportion of AYA achieving minimum cLLDAS as a therapeutic target from 81.9% (*N* = 100) to 91.8% (*N* = 112) over a 12-month period (*P* = 0.022), suggesting that clinician/patient education and co-operation could improve cSLE disease control.

## Conclusions

This prospective study provides the much-needed evidence that T2T strategies are an achievable goal in clinical practice, and that routine objective assessment of disease activity and damage in cSLE could be embedded in the routine clinical consultations, as currently recommended by international guidelines, with the ultimate aim to optimise disease management [22]. Although the SLEDAI-2 K has been recorded in clinical letters in a higher proportion of cases than pBILAG, implementing a consensual treatment target, following agreement with AYA with cSLE and their family/carers, as developmentally appropriate, led to statistically significant improvement in the proportion of individuals able to decrease their steroid treatment dose, as well as of those achieving a better disease control after a medium duration of 12-month routine follow-up. Although this study has not been statistically powered to detect differences in disease control following T2T implementation, the large sample size exceeded the one required to detect a 10% difference in the proportion of AYA with cSLE in target at baseline versus 12 months.

This study provides evidence that agreeing on a treatment target with AYA with cSLE is likely to be applicable in the majority of cases. It also improves awareness that in some selected cSLE cases, discussing and implementing a T2T strategy may be challenging (e.g. following a recent flare) or less achievable despite clinically appropriate, based on individual considerations or disease-related factors (e.g. previous flares following a decrease in medication, exposure to life stressors such as exams, relocating for university or a new job, planning a pregnancy), which may preclude steroid dose optimisation is selected cSLE cases.

## Discussion

This real-life study, evaluating the active implementation of the T2T strategy in routine practice in a large cohort of AYA with cSLE, found a higher proportion of target attainment compared to the UK JSLE cohort [7]. This difference can be explained by various factors: in the present study, all AYA classified as having cSLE attending routine appointments have been evaluated compared to participants with cSLE or evolving cSLE phenotypes consented to take part in research as it is the case with the UK JSLE cohort; differences between the two cSLE cohorts’ demographics and disease duration at inclusion (2 years for the UK JSLE cohort vs. 13.5 years in this study) could have contributed to differences in results; variations in findings derived from a study involving prospective data collection at every routine appointment from a single tertiary centre vs. analysis of retrospective data collected as part of a UK-wide observational study not specifically focused on T2T strategies are to be expected. These aspects ultimately reflect differences in cSLE severity across ages and the lifespan and variation in clinical practice and type of data analysed.

While it is widely recognised that cSLE is more severe than adult SLE at disease onset [3], tighter disease control strategies have been previously assessed in other cSLE cohorts [7, 23, 24]. In a smaller study from the Netherlands, all children with cSLE achieved LLDAS approximately 6 months post-diagnosis [25] through the implementation of a uniform steroid tapering regimen post-diagnosis rather than employing a T2T strategy per se.

More recently, a retrospective analysis of a prospective single-centre cSLE cohort study, with a significantly smaller sample size than the present study, sought to validate the new cLLDAS definition [26]. The study found slightly different time to reach cLLDAS compared to the adult LLDAS definition, used as an argument for cLLDAS validation, and protective effects of maintaining cLLDAS for at least 50% of the time (cLLDAS-50). The cLLDAS-50 status was achieved by 58% of children with cSLE and had significant effects in minimising the damage accumulation [26].

More robust literature findings pertain to adult SLE large multi-centre studies, in which prospective T2T strategy implementation led to improved clinical outcomes, in terms of decreased risk of flare and damage accrual [27, 28]. Additionally, LLDAS achievement was also associated with better quality of life and improvement in pain, fatigue, and overall disease experience [29]. Good disease control in adult SLE has been associated with lower direct and indirect health care costs in the large multi-centre SLICC inception cohort, findings which have significant societal implications [30].

In addition to increasing evidence that T2T strategies are a realistic rather than an aspirational treatment goal in cSLE, constructive efforts have been made by clinicians, charities, and young people to convey the concept of the need for tight disease control in cSLE, which can additionally facilitate its wider clinical implementation [31]. This present study is the first to evaluate the feasibility to implement T2T approaches in routine practice by evaluating AYA with cSLE at each consecutive appointment, which can now reassure the rheumatology community that these strategies can be embedded in clinical practice.

### Study limitations

This is a single-centre study in AYA with longer cSLE disease duration than the retrospective studies previously published, which may not reflect the T2T implementation success in younger paediatric populations characterised by higher cSLE activity at disease onset. Despite being the only large cSLE study which collected data prospectively, the impact of the T2T strategy was not evaluated using a randomised controlled trial design, which would have provided the highest quality of evidence for the significant impact of this strategy on cSLE outcomes. The duration of study follow-up was only 12 months, and therefore, the long-term impact of active T2T strategy implementation will be explored in the future.

In conclusion, this large single-centre prospective study in cSLE project provides the first evidence that proactive implementation of the newly defined T2T outcomes in cSLE is feasible. The success in agreeing with AYA with cSLE a well-defined target at each routine appointment and working together towards achieving it, both led to a significant improvement in disease control and damage accrual over 12 months, providing reassurance that tighter disease control strategies can and should be adopted in routine practice.

## Data Availability

Data can be made available upon request.
